# Cytogenomic characterization of pediatric T-cell acute lymphoblastic leukemia reveals *TCR* rearrangements as predictive factors for exceptional prognosis

**DOI:** 10.1186/s13039-024-00682-4

**Published:** 2024-05-23

**Authors:** Libuse Lizcova, Eva Prihodova, Lenka Pavlistova, Karla Svobodova, Ester Mejstrikova, Ondrej Hrusak, Pavla Luknarova, Iveta Janotova, Lucie Sramkova, Jan Stary, Zuzana Zemanova

**Affiliations:** 1 Center of Oncocytogenomics, Institute of Medical Biochemistry and Laboratory Diagnostics, General University Hospital in Prague and First Faculty of Medicine, Charles University in Prague, Prague, Czech Republic; 2 CLIP - Childhood Leukaemia Investigation Prague, Department of Paediatric Haematology and Oncology, Second Faculty of Medicine, Charles University, Prague and University Hospital Motol, Prague, Czech Republic; 3 Department of Paediatric Haematology and Oncology, Second Faculty of Medicine, Charles University, Prague and University Hospital Motol, Prague, Czech Republic

**Keywords:** Comprehensive cytogenomic analysis, Pediatric T-ALL, *TCR* aberrations, Prognostic factors

## Abstract

**Background:**

T-cell acute lymphoblastic leukemia (T-ALL) represents a rare and clinically and genetically heterogeneous disease that constitutes 10–15% of newly diagnosed pediatric ALL cases. Despite improved outcomes of these children, the survival rate after relapse is extremely poor. Moreover, the survivors must also endure the acute and long-term effects of intensive therapy. Although recent studies have identified a number of recurrent genomic aberrations in pediatric T-ALL, none of the changes is known to have prognostic significance. The aim of our study was to analyze the cytogenomic changes and their various combinations in bone marrow cells of children with T-ALL and to correlate our findings with the clinical features of the subjects and their treatment responses.

**Results:**

We performed a retrospective and prospective comprehensive cytogenomic analysis of consecutive cohort of 66 children (46 boys and 20 girls) with T-ALL treated according to BFM-based protocols and centrally investigated cytogenetics and immunophenotypes. Using combinations of cytogenomic methods (conventional cytogenetics, FISH, mFISH/mBAND, arrayCGH/SNP and MLPA), we identified chromosomal aberrations in vast majority of patients (91%). The most frequent findings involved the deletion of *CDKN2A/CDKN2B* genes (71%), T-cell receptor (*TCR*) loci translocations (27%), and *TLX3* gene rearrangements (23%). All chromosomal changes occurred in various combinations and were rarely found as a single abnormality. Children with aberrations of *TCR* loci had a significantly better event free (*p* = 0.0034) and overall survival (*p* = 0.0074), all these patients are living in the first complete remission. None of the abnormalities was an independent predictor of an increased risk of relapse.

**Conclusions:**

We identified a subgroup of patients with *TCR* aberrations (both *TRA/TRD* and *TRB*), who had an excellent prognosis in our cohort with 5-year EFS and OS of 100%, regardless of the presence of other abnormality or the translocation partner. Our data suggest that escalation of treatment intensity, which may be considered in subsets of T-ALL is not needed for nonHR (non-high risk) patients with TCR aberrations.

## Introduction

T-cell acute lymphoblastic leukemia (T-ALL) is a clinically and genetically heterogeneous disease that constitutes 10–15% of all newly diagnosed pediatric ALL cases [1, 2]. It is slightly more frequent in males than females [2] and is caused by the accumulation of genetic lesions that alter the mechanisms controlling normal T-cell proliferation, differentiation, and survival during thymocyte development [3–5].

Historically, the outcomes for children with T-ALL were inferior to those for children with B-cell acute lymphoblastic leukemia (B-ALL). However, the development of intensified T-ALL-focused protocols has significantly improved the outcomes of these children [6], with a 5-year overall survival rate exceeding 80% [1, 7, 8]. Despite the improved survival rates, ∼ 20% of patients relapse and die owing to acquired therapy resistance. The treatment of relapse is still clinically challenging [9–11], and current efforts are directed primarily toward early identification of high-risk children during treatment and the prevention of recurrent disease. Survivors also endure the acute and long-term effects of intensive toxic chemotherapy. Therefore, it is extremely important to identify those patients who might benefit from reduced-intensity chemotherapy [12].

The cytogenomic abnormalities in pediatric T-ALL are heterogeneous and diverse [1], and they are cryptic on the cytogenetic level in ∼ 50% of cases. The most frequent translocations involve the T-cell receptor (*TCR*) loci *TRA/TRD* (14q11) and *TRB* (7q34), leading to the overexpression of protooncogenes such as *TLX1* (10q24), *TAL1* (1p33), *TAL2* (9q31), *LMO1* (11p15.1), *LMO2* (11p13), *HOXA* (7p15), and *MYC* (8q24). Other chromosomal aberrations lead to the formation of fusion genes encoding abnormal transcription factors. These include the del(1)(p32), t(5;14)(q35;q32), t(10;11)(p13;q14), and 9q34 rearrangements, resulting in *SIL::TAL1*, *TLX3::BCL11B*, *PICALM::MLLT10*, and *NUP214::ABL1* fusions, respectively. Finally, deletions of tumor suppressor loci are commonly found in children with T-ALL, with the deletion of the 9p21 locus (*CDKN2A/CDKN2B* genes) most frequently observed.

Although multiple recurrent genomic aberrations have been identified, none independently predict the outcome of T-ALL and they are not used prospectively in risk stratification [4, 13–17]. Currently, the response to treatment, determined primarily by assessing the minimal residual disease (MRD), is the strongest predictor of outcome for patients with T-ALL [7, 18, 19]. However, most relapses occur in patients who would be predicted to do well based on a favorable MRD response [1], so the search for predictive genetic or clinical markers in pediatric T-ALL is ongoing. Therefore, the aim of our study was to analyze the cytogenomic changes and their various combinations in 66 children with T-ALL treated according to Berlin–Frankfurt–Münster (BFM)-based protocols, and to correlate our findings with the clinical features of the subjects and their treatment responses.

## Materials and methods

### Patients

The bone-marrow samples of consecutive 66 children with T-ALL and their centrally investigated cytogenetics and immunophenotypes were examined in 1996–2017. The clinical characteristics and outcomes of the patients are summarized in Table 1. The study group included 46 boys (69.7%) and 20 girls (30.3%) with a median age of 7.9 years (range 1.1–18.3 years), treated according to BFM-based protocols. Immunophenotypic data classified the patients into pre-T (*n* = 14), cortical-T (*n* = 28), mature-T (*n* = 19), and early-T groups (*n* = 2) based on the European Group for the Immunological Classification of Leukemia (EGIL) criteria [20]. White blood cell (WBC) counts were 3.9–765.1 × 109/L (median 73.5 × 109/L). Forty-two patients were classified as non-high risk (nonHR) and 21 as high risk (HR). Follow-up ranged from 0.3 to 298.6 months (median 86.4 months). Of the 66 patients, 48 are living in first remission (CR1; 45 patients) or second complete remission (CR2; three patients). Relapse occurred in 17 patients and 18 children died. Thirteen patients underwent allogeneic stem-cell transplantation (four of them are living in CR1, three in CR2, and six patients died). Four children developed secondary malignancy (two secondary AML, one secondary MDS, and one glioblastoma). Samples from all patients were analysed with conventional cytogenetic analyses at the time of diagnosis. Retrospective or prospective molecular cytogenomic methods (fluorescence in situ hybridization [FISH]/multicolor FISH [mFISH], multiplex ligation-dependent probe amplification [MLPA], array comparative genomic hybridization/single-nucleotide polymorphism [aCGH/SNP]) were also used to detect cryptic aberrations or complex chromosomal rearrangements. Informed consent for inclusion in the study was obtained from the patients’ parents or guardians.

**Table 1:** Clinical characteristics

	Patients *n* (%)
Sex	
male female	46 (69.7%) 20 (30.3%)
Age at diagnosis (years)	
median range	7.9 1.1–18.3
Immunophenotype	
no further specified early-T pre-T cortical mature	3 (4.5%) 2 (3%) 14 (21.2%) 28 (42.4%) 19 (28.8%)
Risk group	
no data HR non-HR	3 (4.5%) 21 (31.8%) 42 (63.6%)
WBC (109/L)	
median range	73.5 3.9–765.1
Outcome	
CR1 CR2 exitus	45 (68.2%) 3 (4.5%) 18 (27.3%)
Follow up (months)	
median range	86.4 0.3–298.6

^a^HR: high risk; ^b^nonHR: non-high risk; ^c^WBC: white blood cell count; ^d^CR1-first complete remission; ^e^CR2-second complete remission

### Conventional cytogenetics

Bone-marrow samples were cultured for 24 and/or 48 h in MarrowGrow Medium (Cytogen, GmbH, Wetzlar, Germany) without stimulation. Chromosomal preparations were made with standard techniques using colcemid, hypotonic treatment, fixation, and G-banding. Twenty metaphases were analyzed per patient, if available. Chromosomal aberrations were described according to the International System for Human Cytogenomic Nomenclature [21].

### Molecular cytogenetics

To detect the most frequent known chromosomal changes, interphase FISH with commercially available locus-specific probes was used: CytoCell TCRAD Breakapart, CytoCell TCRB (TRB) Breakapart, CytoCell TLX3 Breakapart, and CytoCell TLX1 Breakapart probes (CytoCell, Cambridge, UK), Vysis LSI CDKN2A/CEP 9 FISH Probe Kit, and Vysis LSI BCR/ABL1 DC DF Translocation Probe (Abbott Molecular Diagnostics, Des Plaines, IL, US). At least 200 interphase nuclei were analyzed per probe.

An MLPA analysis with SALSA MLPA Probemix ALL-IKZF1 (MRC-Holland, Amsterdam, the Netherlands) was used to detect deletions and amplifications of the *IKZF1*, *CDKN2A/CDKN2B*, *PAX5*, *ETV6*, *BTG1*, and *RB1* genes when fixed material was available for DNA isolation (50/66 cases).

Complex chromosomal rearrangements were confirmed with multicolor FISH and multicolor banding using 24*X*Cyte and *X*Cyte color kits and an ISIS computer analysis system (MetaSystems, Altlussheim, Germany). In some cases, the aCGH/SNP technique, with the Sure Print G3 Cancer CGH + SNP 4 × 180 K Microarray (Agilent Technologies, Santa Clara, CA, US), was used to detect copy number changes.

### Statistical analysis

Differences in overall survival (OS) and event-free survival (EFS) were assessed using the Kaplan–Meier method and the Mantel–Cox test. EFS was calculated as the time between the date of diagnosis and the date of any event that was defined as “relapse” or “second neoplasm” or “death”, whichever occurred first. Cases with no event were censored at the date of the last follow-up. OS was measured from diagnosis to death or the last follow-up.

## Results

### Cytogenomic analyses

Samples of all 66 children were analyzed using conventional cytogenetic analyses. Informative karyotypic results were obtained in 58 (88%) cases, and 36 of these 58 subjects (62%) were cytogenetically abnormal. Structural aberrations were found in most of these patients, whereas numerical abnormalities were detected in only four cases. Tetraploidy was confirmed in two patients and complex karyotypes (including three or more aberrations) in seven cases.

Using FISH and other cytogenomic methods, chromosomal aberrations were detected in 60/66 (91%) patients. The most frequent aberrations were deletions of the *CDKN2A* gene (in 47 [71%] patients), rearrangements of *TCR* loci (*TRA/TRD* and/or *TRB*) in 16 (27%) children, and *TLX3* gene rearrangements in 15 (23%) cases. Translocations involving the *TRA/TRD* locus were demonstrated in nine cases. In five of these patients, known recurrent chromosomal translocations were detected: t(8;14)(q24;q11), t(11;14)(p13;q11), t(1;14)(p32;q11), and t(10;14)(q24;q11)x2, involving the oncogenes *MYC* (8q24), *LMO2* (11p13), *TAL1* (1p32), and *TLX1* (10q24), respectively. In the remaining four cases, the *TRA/TRD* rearrangements were cryptic. Of the five patients with rearrangements at the *TRB* locus, the partner chromosomes and oncogenes were identified in three cases: ins(1;7)(p32;q34q34), t(7;11)(q34;p13), and t(7;9)(q34;q32), affecting *TAL1* (1p32), *LMO2* (11p13), and *TAL2* (9q31) gene, respectively. The simultaneous occurrence of *TRA/TRD* and *TRB* translocations was detected in two patients, in one of whom translocations t(1;7)(p32;q34) and t(11;14)(p13;q11) were found. In the second case, the *TCR* aberrations were part of a complex karyotype. The commonest fusion involving the *TLX3* gene (*BCL11B::TLX3*), resulting from translocation t(5;14)(q35;q32), was detected in two patients. In the remaining 12 patients with a *TLX3* gene rearrangement, the chromosomal partner was not identified because these aberrations were cryptic.

**Fig. 1:**
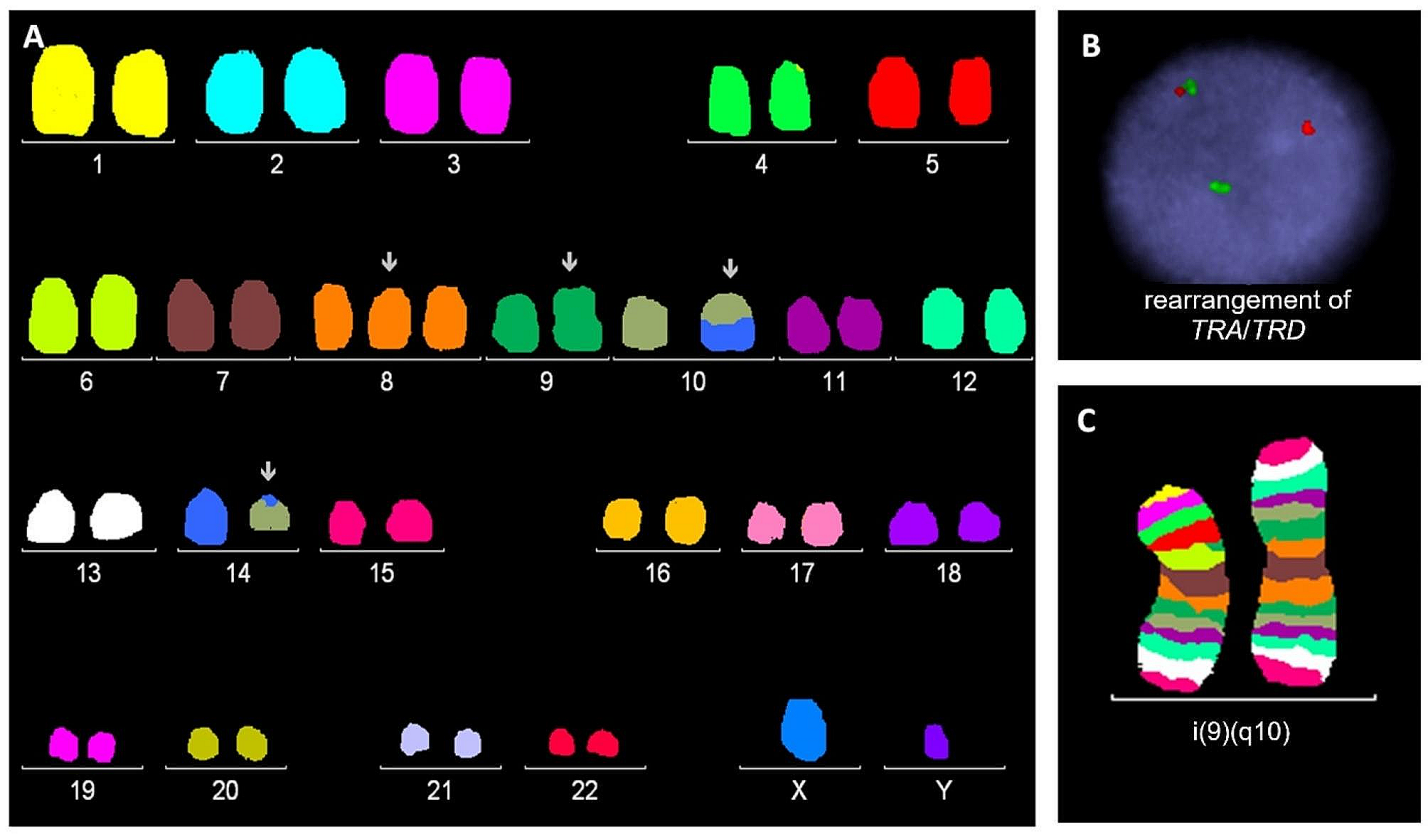
Complex karyotype analyzed by mFISH (**A**), I-FISH: CytoCell TCRAD Breakapart Probe (**B**) and mBAND (**C**): 47,XY,+8,i(9)(q10),t(10;14)(q24;q11)

**Fig. 2:**
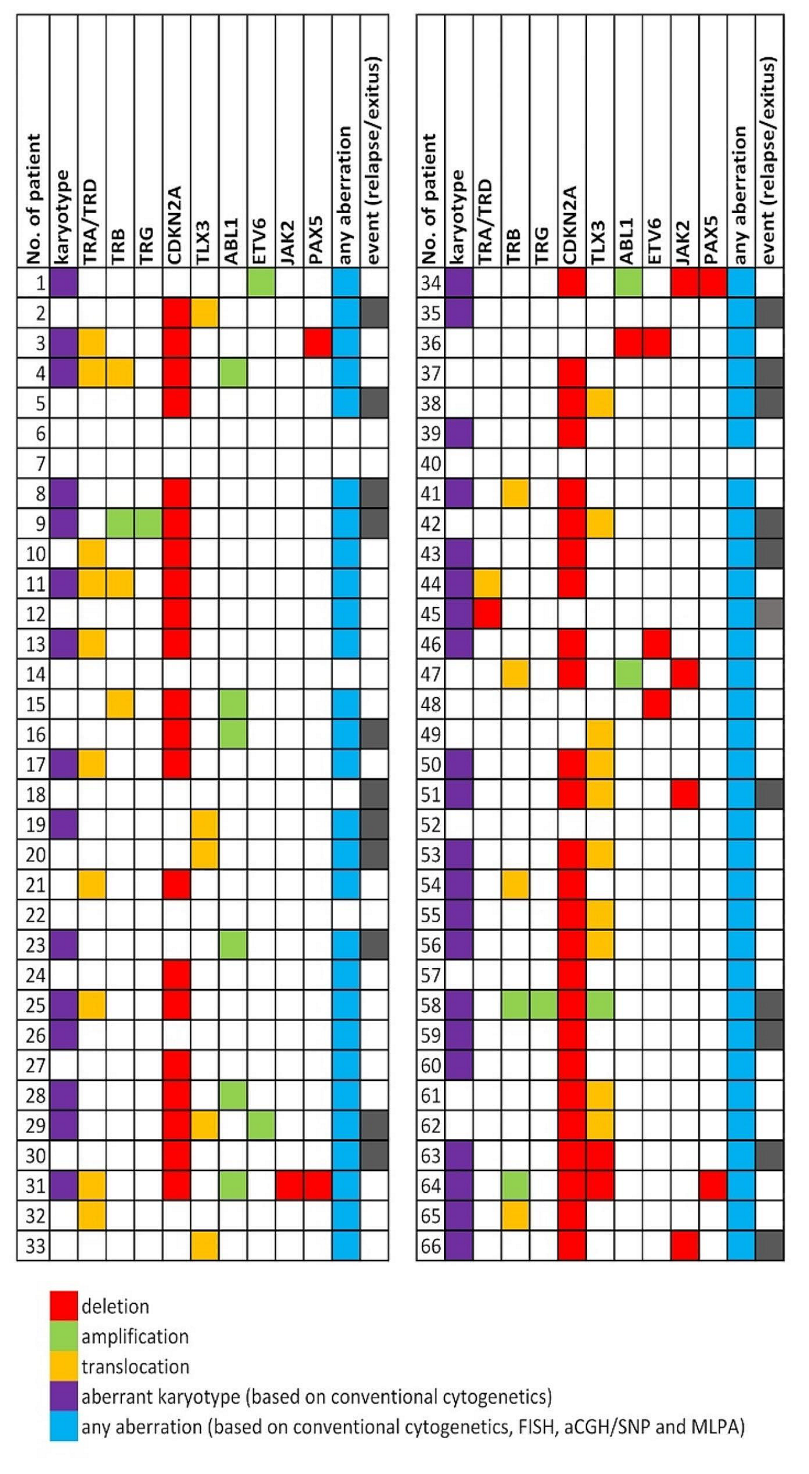
Cytogenomic profiles

Other recurrent aberrations included deletions or amplifications involving the *ABL1* (9q34) gene (8x), *JAK2* (9p24) gene (5x), *PAX5* (9p13) gene (4x), and *ETV6* (12p13) gene (5x). In two of these cases, an isochromosome of the long arm of chromosome 9 was detected, leading to the loss of 9p and the amplification of 9q. Karyotype of this patient analyzed by cytogenomic methods is shown in Fig. 1. In one case, a large deletion of the short arm of chromosome 12 was detected with conventional cytogenetic methods. In the remaining patients, the rearrangements of these genes were cryptic. The most frequent aberrations and their types are summarized in Fig. 2.

**Fig. 3:**
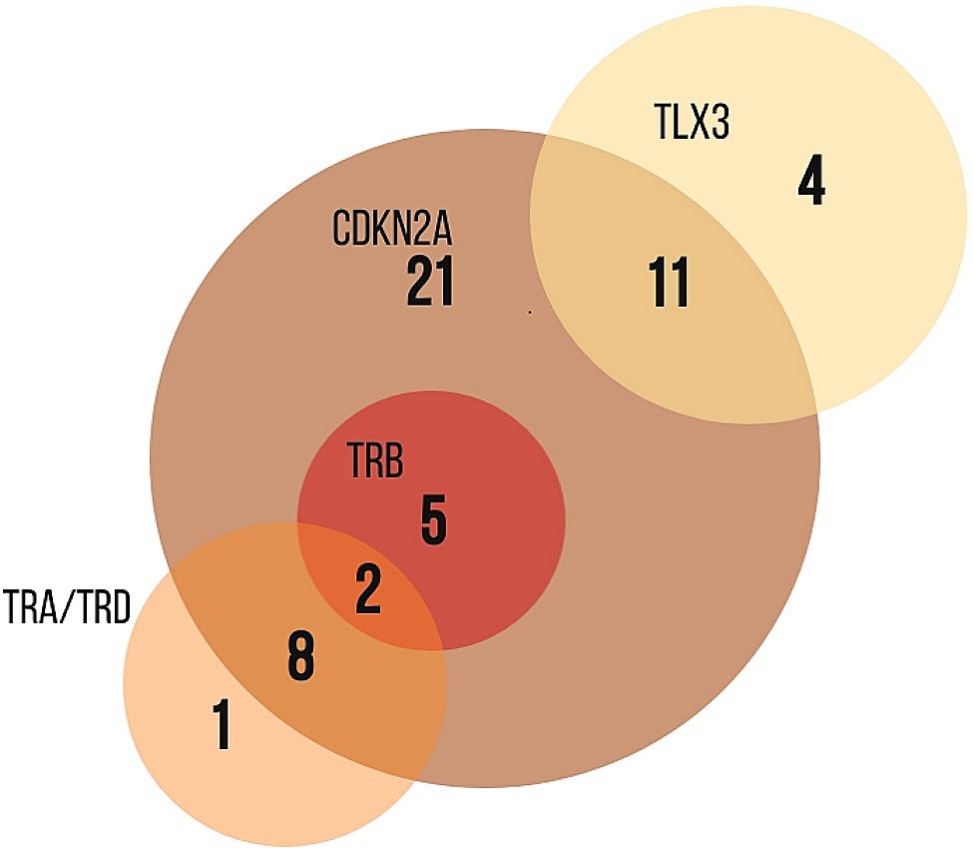
The Venn diagram showing combinations of the most frequent aberrations

All chromosomal changes occurred in various combinations and were rarely found as a single abnormality. However, *TLX3* aberrations never appeared with rearrangements at *TCR* loci. Combinations of the most frequent aberrations are show in Fig. 3.

### Clinical features and outcomes

The clinical characteristics of the patients, stratified according to the results of conventional cytogenetics and the most frequent chromosomal aberrations detected with cytogenomic methods, are summarized in Table 2. The level of MRD determined by *IgH/TCR* rearrangements with reverse transcription polymerase chain reaction (RT-PCR) is not shown as it was not available for all patients (MRD measurement using *IgH/TCR* rearrangements was not involved in older treatment protocols). There were no significant differences among the various cytogenetic subgroups and clinical factors such as sex, age, or WBC count. However, all children with rearrangements at *TCR* loci (*TRA/TRD* and *TRB*) were nonHR patients with cortical or mature immunophenotypes.

**Table 2:** Clinical data and outcomes according to the most frequent chromosomal aberrations

	TLX3 *n* = 15/66	CDKN2A *n* = 47/66	TCR *n* = 16/66	^f^cyto aberrant *n* = 29/66	^g^cyto complex *n* = 7/66	^h^cyto nn *n* = 22/66
Sex						
male female	12 3	34 13	11 5	22 7	4 3	16 6
Age at diagnosis						
median range	6.2 4.6–16.5	8.6 1.1–18.3	11.2 3.3–18.3	7.7 1.1–18.3	5.8 1.1–16.4	7.6 3.8–17.8
Immunophenotype						
no further specified early-T pre-T cortical mature	1 - 4 8 2	2 1 7 21 15	1 - - 10 5	2 1 7 10 9	- - 1 6 0	- 1 4 9 8
Risk group						
no data ^a^HR ^b^nonHR	- 7 8	3 11 33	1 - 15	3 9 17	- 1 6	- 10 12
^c^WBC (109/L)						
median range	81.2 6.8-765.1	96.1 4.7-765.1	37.45 3.9–511.0	96.1 5.8-765.1	54.1 16.0-205.0	71.6 3.9–726
Event	7	15	-	10	3	7
Exitus	7	12	-	8	3	6
5-year ^d^OS (^e^SE) %	51.4 (13.4)	73.4 (6.63)	100.0 (-)	70.0 (6.13)	57.1 (18.7)	72.1 (9.71)

^a^HR: high risk; ^b^nonHR: non-high risk; ^c^WBC: white blood cell count; ^d^OS: overall survival; ^e^SE: standard error; ^f^cyto aberrant: one or two chromosomal aberrations; ^g^cyto complex: three or more chromosomal aberrations; ^h^cyto nn: normal karyotype

**Fig. 4:**
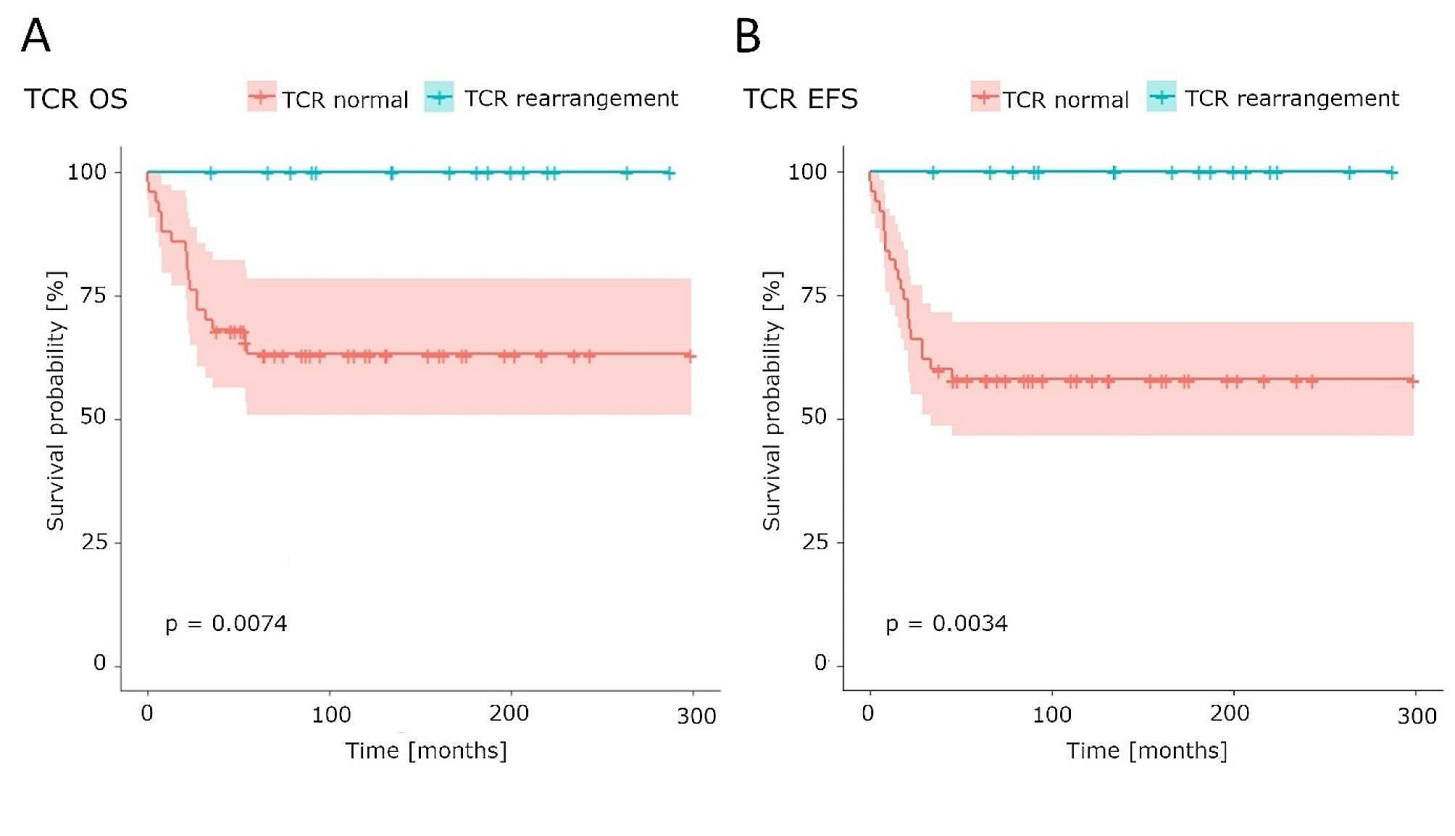
Overall (**A**) and event free (**B**) survival of patients with (red) and without (green) *TCR* rearrangements

The associations between EFS or OS and particular cytogenomic aberrations were analyzed. The patients were divided into three groups based on conventional karyotyping: normal karyotype, abnormal karyotype (one or two aberrations), or complex karyotype (three or more aberrations). There were no significant differences between the various cytogenetic subgroups in EFS or OS. According to the commonest chromosomal abnormalities found with cytogenomic methods (*TCR* loci rearrangements, *CDKN2A* deletions, and *TLX3* translocations), the patients were stratified into group with or without the aberration. Significantly better EFS (*p* = 0.0034) and OS (*p* = 0.0074) were observed in children with *TCR* locus rearrangements (*TRA/TRD* and/or *TRB*), Fig. 4. There were no events in this group of patients. An analysis of *CDKN2A* deletions and *TLX3* aberrations showed that neither EFS nor OS differed significantly in patients with and without these abnormalities (*CDKN2A* pEFS = 0.81 and *CDKN2A* pOS = 0.8; *TLX3* pEFS = 0.46 and *TLX3* pOS = 0.19). Because all patients with *TCR* rearrangements were nonHR, a separate survival analysis of these risk groups was performed. There was no significant difference in EFS between the HR and nonHR patients (*p* = 0.22). However, in the overall survival analysis, a difference was detected at the cut-off level for significance (*p* = 0.054).

## Discussion

Recent studies of T-ALL biology based on contemporary cytogenomic assays have identified a number of recurrent lesions and clearly improved the characterization of pediatric T-ALL. However, these have altered neither risk stratification nor treatment because none of the identified changes is known to have prognostic significance under current treatment protocols [22, 23]. In the present study, we performed a comprehensive cytogenomic analysis of 66 children with T-ALL, and we correlated our findings with the clinical data and treatment responses. We identified the chromosomal alterations in the vast majority of patients (91%). As expected, the commonest findings involved the deletion of the *CDKN2A/CDKN2B* genes (71%), *TCR* locus translocations (27%), and *TLX3* gene rearrangements (23%). Patients in our cohort with *TCR* aberrations, either *TRA/TRD* or *TRB*, had excellent prognoses (pEFS = 0.0034 and pOS = 0.0074, with 5-year OS 100%). The clinical characteristics of the patients were consistent with those of previous larger series, with a median age of 7.9 years, male predominance, and a median WBC count of 73.5 × 109/L [24].

The range of genetic abnormalities in pediatric T-ALL is diverse, and multiple oncogenes and tumor suppressor genes cooperate to alter the mechanisms controlling normal T-cell development. Despite this significant heterogeneity, most aberrations can be classified into two distinct categories. The first includes chromosomal translocations that arrest T-cell development at a specific maturation stage and are associated with a distinct gene expression signature. The second category includes deletions and mutations that affect signaling and/or cell-cycle pathways and are often present in combination with chromosomal translocations [4, 16, 25, 26].

The first group includes rearrangements that juxtapose *TCR* genes and protooncogenes that encode pivotal transcription factors. These aberrations are the main factors initiating the events in T-ALL carcinogenesis, whereas gene mutations and deletions are secondary and are important in clonal evolution and overt leukemia [24]. Consistent with previously published data [2, 24, 27], *TCR* rearrangements were the commonest chromosomal translocations in this series, detected in 27% of patients. The *TRA/TRD* and *TRB* genes were affected in nine and five patients, respectivelyA rare simultaneous occurrence of rearrangements at the *TRA/TRD* and *TRB* loci in one pathological clone was detected in two patients and both *TCR* genes targeted different T-cell oncogenes in these cases. Cauwelier et al. [28] have shown that as many as half of all *TRB* rearrangements and about one third of *TRA/TRD* rearrangements are not detected in karyotype analyses. Altogether, we did not detect the *TCR* aberrations with conventional karyotyping in seven patients. This could be explained by the distal localization of the breakpoint loci on the partner chromosomes (especially for the *TRB* rearrangements) or by the presence of complex karyotypes in these cases. We suggest that nonleukemic cells were cultivated in some patients because these aberrations were only detected in interphase nuclei. Given the published data, the prognostic significance of *TCR* rearrangements is generally unclear. However, according to some authors, the prognosis depends on translocation partner [29]. In most studies, aberrations involving the *TLX1* and *TAL1* genes were associated with favorable outcome [14, 29–32], while the prognosis of other *TCR* translocations was unknown [17, 32–34]. Nevertheless, according to Nordic Study [24] of 285 pediatric T-ALL cases, rare *TCR* aberrations including t(X;14)(p11;q11), t(X;7)(q22;q34), t(1;14)(p32;q11), ins(14;5)(q11;q? q?), inv(7)(p15q34), t(8;14)(q24;q11), t(7;11)(q34;p15) and t(12;14)(p13;q11), were associated with poor prognosis. Although we found two of these aberrations in our cohort, i.e. t(1;14)(p32;q11) and t(8;14)(q24;q11), all *TCR* rearrangements were associated with clinically favorable outcome (5-year OS of 100%) in this study.

The other common translocations included the *TLX3* gene, a homeobox gene that is not expressed in normal T-cell development [33]. These aberrations occur in around 20–25% of T-ALL, and the *TLX3* gene is aberrantly activated by various cryptic translocations, including t(5;14)(q35;q32), which juxtapose *TLX3* with *BCL11B*, a gene expressed during T-cell maturation [25, 34, 35]. Consistent with these findings, we detected *TLX3* gene aberrations in 23% of the patients and demonstrated t(5;14) in two of them. Some alternative *TLX3* aberrations have been described involving the *TRA/TRD* locus [36]. However, in our cohort, no *TLX3* rearrangements involving *TCR* loci were detected, confirming the very rare occurrence of this abnormality (˂1% of cases). Conflicting data have been reported about *TLX3* rearrangements and prognosis. In some studies, patients with *TLX3* aberrations had a poor prognosis, with 3-year survival rates of 45–50% [32, 37], whereas in other studies, *TLX3* translocations were associated with an improved outcome or had no effect on the prognosis [5, 30, 38]. In our series, the 5-year EFS was 51.4%, which was the lowest EFS of all cytogenomic categories compared (see Table 2.). However, it did not differ significantly from that of patients without *TLX3* rearrangements (*p* = 0.19). The data for *TLX3*-positive T-ALL leukemias are clearly variable, possibly due to differences in treatment protocols, the sizes of cohorts, or the presence of additional genetic abnormalities.

In agreement with other studies [17, 27, 39, 40], *CDKN2A/CDKN2B* deletions were the most frequent cytogenomic aberrations detected (in 71% of patients). These genes exert a tumor suppressor effect, and their inactivation leads to uncontrolled neoplastic proliferation. We detected homozygous (biallelic) deletionsin 30 of 47 patients. In the remaining 17 children, in whom the heterozygous (monoallelic) deletion was found, the inactivation of the second allele was probably by mutation or epigenetic silencing by hypermethylation of the promoter, as previously described [41, 42]. The prognostic value of the *CDKN2A/CDKN2B* gene deletion and its association with some clinical features are highly contentious. Some authors have described a significantly lower survival rate, older age at diagnosis, or higher WBC count in patients with this deletion. However, other studies as well as ours have reported that the prognosis of patients with this deletion is unclear [42–44]. These cases probably formed a heterogeneous group of patients, with a number of other chromosomal aberrations. Moreover, the deletion of these genes is not restricted to T-cell leukemia. Therefore, we assume that *CDKN2A/CDKN2B* aberrations reflect a general mechanism of cancer rather than a specific prognostic group of children with T-ALL.

A wide spectrum of deletions and amplifications of genes affecting crucial signaling pathways has already been described in pediatric T-ALL [13]. We detected the gain of the *ABL1* gene in eight patients. Surprisingly, we detected no *ABL1::NUP214* fusion in our cohort, which is described in ∼ 5% of T-ALL patients [45]. However, the assessment of *ABL1* rearrangements in pediatric T-ALL is important because treatment with tyrosine kinase inhibitors is possible [22, 45]. We identified deletions of the transcription factor genes *ETV6* and *PAX5* in five and four patients, respectively. Although these genes are involved primarily in B-cell proliferation and differentiation, consistent with other studies, we suggest that the loss of their functions also plays a significant role in T-ALL [7, 46]. Advances in next-generation sequencing (NGS) have identified mutations in several genes that also play a significant role in the pathogenesis of T-ALL and could be used for stratifying patients with this rare and genetically heterogeneous disease. In particular, the mutational status of *PI3K, NOTCH, FBXW7, PTEN, KRAS* and *RAS* genes is likely to be of prognostic significance and could update risk classification [15, 17, 47, 48]. Although sequencing data are not available in this retrospective study, integrating comprehensive genomic testing, including chromosomal aberrations and mutational profiles, is important for future research studies and subsequent risk stratification and tailoring of treatment options. We detected cytogenomic aberrations in the vast majority of children in our cohort and showed that they occurred more often in various combinations than individually, confirming the multistep process of T-ALL pathogenesis. It is assumed that chromosomal aberrations initiate the process of carcinogenesis, but without accompanying copy number changes, they are not responsible for the formation of leukemic cells [13]. In our cohort, all changes occurred in various combinations, although *TLX3* rearrangements never occurred with *TCR* locus abnormalities. This corresponded to the different survival rates of patients with these two aberrations in our study. By contrast, the *CDKN2A* gene was deleted in all patients with *PAX5* and *JAK2* gene deletions, which is consistent with other studies that have shown the statistically significant co-occurrence of these deletions [7, 49, 50].

## Conclusion

In conclusion, we have demonstrated that pediatric T-ALL represents a rare and highly genetically heterogeneous disease. Although the outcomes of these children have improved significantly in recent decades, the survival rate after relapse is still extremely poor. Moreover, the survivors must also endure the acute and long-term effects of intensive toxic chemotherapy. Because there is no reliable biomarker to identify these groups of patients at the time of diagnosis, we used comprehensive cytogenomic techniques to analyze bone marrow samples of 66 children with T-ALL. In almost all patients, we found a number of genetic aberrations that occurred in various combinations. However, none of these abnormalities was an independent predictor of an increased risk of relapse. Nevertheless, we identified a subgroup of patients with *TCR* aberrations (both *TRA/TRD* and *TRB*), who had an excellent prognosis in our cohort (5-year OS of 100%). We hypothesize that escalation of treatment intensity, which may be considered in subsets of T-ALL is not needed for nonHR patients with *TCR* aberrations.

## Data Availability

No datasets were generated or analysed during the current study.

## References

[1] Raetz EA, Teachey DT (2016). T-cell acute lymphoblastic leukemia. Hematol Am Soc Hematol Educ Program.

[2] Chiaretti S, Gianfelici V, O’Brien SM, Mullighan CG (2016). Advances in the Genetics and Therapy of Acute Lymphoblastic Leukemia. Am Soc Clin Oncol Educ Book.

[3] Girardi T, Vicente C, Cools J, De Keersmaecker K (2017). The genetics and molecular biology of T-ALL. Blood.

[4] Hefazi M, Litzow MR (2018). Recent advances in the Biology and Treatment of T Cell Acute Lymphoblastic Leukemia. Curr Hematol Malig Rep.

[5] Van Vlierberghe P, Pieters R, Beverloo HB, Meijerink JP (2008). Molecular-genetic insights in paediatric T-cell acute lymphoblastic leukaemia. Br J Haematol.

[6] Teachey DT, O’Connor D (2020). How I treat newly diagnosed T-cell acute lymphoblastic leukemia and T-cell lymphoblastic lymphoma in children. Blood.

[7] Lejman M, Włodarczyk M, Styka B (2020). Advantages and limitations of SNP array in the Molecular characterization of Pediatric T-Cell Acute Lymphoblastic Leukemia. Front Oncol.

[8] Winter SS, Dunsmore KP, Devidas M, et al. Improved survival for children and young adults with T-Lineage Acute Lymphoblastic Leukemia: results from the children’s Oncology Group AALL0434 methotrexate randomization [published correction appears in J Clin Oncol. 2019;37(9):761]. J Clin Oncol. 2018;36(29):2926–34. 10.1200/JCO.2018.77.7250 PMC6366301 30138085

[9] Pui CH, Pei D, Cheng C (2019). Treatment response and outcome of children with T-cell acute lymphoblastic leukemia expressing the gamma-delta T-cell receptor. Oncoimmunology.

[10] Eckert C, Parker C, Moorman AV (2021). Risk factors and outcomes in children with high-risk B-cell precursor and T-cell relapsed acute lymphoblastic leukaemia: combined analysis of ALLR3 and ALL-REZ BFM 2002 clinical trials. Eur J Cancer.

[11] Hunger SP, Raetz EA (2020). How I treat relapsed acute lymphoblastic leukemia in the pediatric population. Blood.

[12] Schwab C, Harrison CJ (2018). Advances in B-cell precursor Acute Lymphoblastic Leukemia Genomics. Hemasphere.

[13] Mroczek A, Zawitkowska J, Kowalczyk J, Lejman M (2021). Comprehensive Overview of Gene rearrangements in childhood T-Cell Acute Lymphoblastic Leukaemia. Int J Mol Sci.

[14] Olshanskaya Y, Kazakova A, Tsaur G (2019). Clinical significance of cytogenetic changes in childhood T-cell acute lymphoblastic leukemia: results of the multicenter group Moscow-Berlin (MB). Leuk Lymphoma.

[15] Burns MA, Place AE, Stevenson KE, et al. Identification of prognostic factors in childhood T-cell acute lymphoblastic leukemia: results from DFCI ALL Consortium protocols 05 – 001 and 11 – 001 [published correction appears in Pediatr Blood Cancer. 2021;68(3):e28885]. Pediatr Blood Cancer. 2021;68(1):e28719. 10.1002/pbc.28719 PMC8369809 33026184

[16] Teachey DT, Pui CH (2019). Comparative features and outcomes between paediatric T-cell and B-cell acute lymphoblastic leukaemia. Lancet Oncol.

[17] Liu Y, Easton J, Shao Y (2017). The genomic landscape of pediatric and young adult T-lineage acute lymphoblastic leukemia. Nat Genet.

[18] Schrappe M, Valsecchi MG, Bartram CR (2011). Late MRD response determines relapse risk overall and in subsets of childhood T-cell ALL: results of the AIEOP-BFM-ALL 2000 study. Blood.

[19] Karrman K, Johansson B (2017). Pediatric T-cell acute lymphoblastic leukemia. Genes Chromosomes Cancer.

[20] Bene MC, Castoldi G, Knapp W (1995). Proposals for the immunological classification of acute leukemias. European Group for the Immunological Characterization of Leukemias (EGIL). Leukemia.

[21] McGowan-Jordan J, Hastings RJ, Moore S (2020). ISCN 2020: an International System for Human Cytogenomic nomenclature.

[22] Peterson JF, Pitel BA, Smoley SA (2019). Detection of a cryptic NUP214/ABL1 gene fusion by mate-pair sequencing (MPseq) in a newly diagnosed case of pediatric T-lymphoblastic leukemia. Cold Spring Harb Mol Case Stud.

[23] Taylor J, Xiao W, Abdel-Wahab O (2017). Diagnosis and classification of hematologic malignancies on the basis of genetics. Blood.

[24] Karrman K, Forestier E, Heyman M (2009). Clinical and cytogenetic features of a population-based consecutive series of 285 pediatric T-cell acute lymphoblastic leukemias: rare T-cell receptor gene rearrangements are associated with poor outcome. Genes Chromosomes Cancer.

[25] Patrick K, Vora A (2015). Update on biology and treatment of T-cell acute lymphoblastic leukaemia. Curr Opin Pediatr.

[26] Belver L, Ferrando A (2016). The genetics and mechanisms of T cell acute lymphoblastic leukaemia. Nat Rev Cancer.

[27] Graux C, Cools J, Michaux L, Vandenberghe P, Hagemeijer A (2006). Cytogenetics and molecular genetics of T-cell acute lymphoblastic leukemia: from thymocyte to lymphoblast. Leukemia.

[28] Cauwelier B, Cavé H, Gervais C (2007). Clinical, cytogenetic and molecular characteristics of 14 T-ALL patients carrying the TCRbeta-HOXA rearrangement: a study of the Groupe Francophone De Cytogénétique Hématologique. Leukemia.

[29] Szczepański T, Harrison CJ, van Dongen JJ (2010). Genetic aberrations in paediatric acute leukaemias and implications for management of patients. Lancet Oncol.

[30] Cavé H, Suciu S, Preudhomme C (2004). Clinical significance of HOX11L2 expression linked to t(5;14)(q35;q32), of HOX11 expression, and of SIL-TAL fusion in childhood T-cell malignancies: results of EORTC studies 58881 and 58951. Blood.

[31] Bergeron J, Clappier E, Radford I (2007). Prognostic and oncogenic relevance of TLX1/HOX11 expression level in T-ALLs. Blood.

[32] van Grotel M, Meijerink JP, Beverloo HB (2006). The outcome of molecular-cytogenetic subgroups in pediatric T-cell acute lymphoblastic leukemia: a retrospective study of patients treated according to DCOG or COALL protocols. Haematologica.

[33] Van Vlierberghe P, Homminga I, Zuurbier L (2008). Cooperative genetic defects in TLX3 rearranged pediatric T-ALL. Leukemia.

[34] Meijerink JP, den Boer ML, Pieters R (2009). New genetic abnormalities and treatment response in acute lymphoblastic leukemia. Semin Hematol.

[35] Bernard OA, Busson-LeConiat M, Ballerini P (2001). A new recurrent and specific cryptic translocation, t(5;14)(q35;q32), is associated with expression of the Hox11L2 gene in T acute lymphoblastic leukemia. Leukemia.

[36] Hansen-Hagge TE, Schäfer M, Kiyoi H (2002). Disruption of the RanBP17/Hox11L2 region by recombination with the TCRdelta locus in acute lymphoblastic leukemias with t(5;14)(q34;q11). Leukemia.

[37] Ballerini P, Landman-Parker J, Cayuela JM (2008). Impact of genotype on survival of children with T-cell acute lymphoblastic leukemia treated according to the French protocol FRALLE-93: the effect of TLX3/HOX11L2 gene expression on outcome. Haematologica.

[38] Attarbaschi A, Pisecker M, Inthal A (2010). Prognostic relevance of TLX3 (HOX11L2) expression in childhood T-cell acute lymphoblastic leukaemia treated with Berlin-Frankfurt-Münster (BFM) protocols containing early and late re-intensification elements. Br J Haematol.

[39] Richter-Pechańska P, Kunz JB, Hof J (2017). Identification of a genetically defined ultra-high-risk group in relapsed pediatric T-lymphoblastic leukemia. Blood Cancer J.

[40] La Starza R, Pierini V, Pierini T (2020). Design of a comprehensive fluorescence in situ hybridization assay for genetic classification of T-Cell Acute Lymphoblastic Leukemia. J Mol Diagn.

[41] Herman JG, Jen J, Merlo A, Baylin SB (1996). Hypermethylation-associated inactivation indicates a tumor suppressor role for p15INK4B. Cancer Res.

[42] Agarwal M, Bakhshi S, Dwivedi SN, Kabra M, Shukla R, Seth R (2018). Cyclin dependent kinase inhibitor 2A/B gene deletions are markers of poor prognosis in Indian children with acute lymphoblastic leukemia. Pediatr Blood Cancer.

[43] Karrman K, Castor A, Behrendtz M (2015). Deep sequencing and SNP array analyses of pediatric T-cell acute lymphoblastic leukemia reveal NOTCH1 mutations in minor subclones and a high incidence of uniparental isodisomies affecting CDKN2A. J Hematol Oncol.

[44] Genescà E, Lazarenkov A, Morgades M (2018). Frequency and clinical impact of CDKN2A/ARF/CDKN2B gene deletions as assessed by in-depth genetic analyses in adult T cell acute lymphoblastic leukemia. J Hematol Oncol.

[45] Graux C, Stevens-Kroef M, Lafage M (2009). Heterogeneous patterns of amplification of the NUP214-ABL1 fusion gene in T-cell acute lymphoblastic leukemia. Leukemia.

[46] Jung M, Schieck M, Hofmann W (2020). Frequency and prognostic impact of PAX5 p.P80R in pediatric acute lymphoblastic leukemia patients treated on an AIEOP-BFM acute lymphoblastic leukemia protocol. Genes Chromosomes Cancer.

[47] Fogelstrand L, Staffas A, Wasslavik C (2014). Prognostic implications of mutations in NOTCH1 and FBXW7 in childhood T-ALL treated according to NOPHO ALL-1992 and ALL-2000 protocols. Pediatr Blood Cancer.

[48] Paganin M, Grillo MF, Silvestri D (2018). The presence of mutated and deleted PTEN is associated with an increased risk of relapse in childhood T cell acute lymphoblastic leukaemia treated with AIEOP-BFM ALL protocols. Br J Haematol.

[49] Den Boer ML, van Slegtenhorst M, De Menezes RX (2009). A subtype of childhood acute lymphoblastic leukaemia with poor treatment outcome: a genome-wide classification study. Lancet Oncol.

[50] Mullighan CG, Goorha S, Radtke I (2007). Genome-wide analysis of genetic alterations in acute lymphoblastic leukaemia. Nature.

